# Therapeutic Strategies Targeting DUX4 in FSHD

**DOI:** 10.3390/jcm9092886

**Published:** 2020-09-07

**Authors:** Laura Le Gall, Eva Sidlauskaite, Virginie Mariot, Julie Dumonceaux

**Affiliations:** 1 NIHR Biomedical Research Centre, University College London, Great Ormond Street Institute of Child Health and Great Ormond Street Hospital NHS Trust, London WC1N 1EH, UK; l.gall@ucl.ac.uk (L.L.G.); e.sidlauskaite@ucl.ac.uk (E.S.); virginie.mariot@ucl.ac.uk (V.M.); 2 Northern Ireland Center for Stratified/Personalised Medicine, Biomedical Sciences Research Institute, Ulster University, Derry~Londonderry, Northern Ireland BT47 6SB, UK

**Keywords:** FSHD, DUX4, therapy, animal models, facioscapulohumeral dystrophy, muscle

## Abstract

Facioscapulohumeral muscular dystrophy (FSHD) is a common muscle dystrophy typically affecting patients within their second decade. Patients initially exhibit asymmetric facial and humeral muscle damage, followed by lower body muscle involvement. FSHD is associated with a derepression of *DUX4* gene encoded by the D4Z4 macrosatellite located on the subtelomeric part of chromosome 4. DUX4 is a highly regulated transcription factor and its expression in skeletal muscle contributes to multiple cellular toxicities and pathologies ultimately leading to muscle weakness and atrophy. Since the discovery of the FSHD candidate gene *DUX4*, many cell and animal models have been designed for therapeutic approaches and clinical trials. Today there is no treatment available for FSHD patients and therapeutic strategies targeting DUX4 toxicity in skeletal muscle are being actively investigated. In this review, we will discuss different research areas that are currently being considered to alter *DUX4* expression and toxicity in muscle tissue and the cell and animal models designed to date.

## 1. Introduction

Facioscapulohumeral muscular dystrophy (FSHD, OMIM: 158900, 158901) is one of the most common hereditary muscular dystrophies after Duchenne (DMD) and myotonic dystrophies. Most cases of FSHD are inherited in an autosomal dominant manner. The first muscles clinically affected by the disease are those of the face and shoulders and symptoms often progress towards the lower body affecting humeral, truncal, and leg muscles (for review see [1]). Affected muscles display different histopathological signs easily detected in muscle biopsy samples, including apoptosis [2] and inflammatory infiltration including T- and B-cells and macrophages [3,4]. 

The genetic and epigenetic mechanisms underlying FSHD pathology have been deciphered over the past decades and the chromosomal rearrangement at the 4q subtelomeric region was first localized in the early 90s [5]. The D4Z4 repeat consists of repeated 3.3 kb identical units distinguished by the KpnI restriction enzyme. The vast majority of FSHD patients present 1–10 D4Z4 units whereas healthy individuals usually carry 11 to 100 D4Z4 repeats [6]. However, the genetics of FSHD is complex and exceptions have been described to this general rule with 1–3% of normal individuals having presented a reduced number of D4Z4 repeats [7,8]. In each D4Z4 repeat, several genetic elements were identified. A TACAA-included promoter was located upstream of a potential open reading frame element [9] containing two homeodomain sequences. Each D4Z4 repeat contains one copy of the double homeobox 4 (*DUX4*) gene [9], but only the last repeat in the subtelomeric region of chromosome 4q encodes a functional DUX4 due to the presence of a downstream polyadenylation site located outside of the repeat, in the pLAM region [10], which is present only on the 4qA haplotype [11]. Studies have demonstrated that *DUX4* mRNAs undergo alternate splicing in muscle [10,12]: Three *DUX4* isoforms are mainly produced, a truncated DUX4 known as *DUX4-s*, and two full length *DUX4* transcripts, *DUX4-fl*. *DUX4-fl* transcripts code the full length DUX4 protein, whereas *DUX4*-s codes a truncated and inactive version of DUX4 and therefore *DUX4-fl* transcripts are the only transcript associated with FSHD pathology (for review see [13]). Indeed, DUX4 is a toxic transcription factor, activating hundreds of downstream genes, impairing muscle development, activating the immune response, increasing susceptibility to oxidative stress, ultimately leading to the activation of cell death pathways (for review see [14]). 

Among all patients with FSHD clinical signs, only 95% will present a D4Z4-repeat contraction known as FSHD1. The high level of hypomethylation [15] associated with an opened chromatin structure due to repeat-contraction, leads to the derepression of *DUX4* in skeletal muscle and the progression of clinical FSHD symptoms. This *DUX4* expression is also observed in the remaining 5% of FSHD patients (termed FSHD2) who, unlike FSHD1 patients, present a demethylation of the D4Z4 array of both alleles of chromosomes 4 and 10 [15,16] due to mutations in epigenetic repressors including the structural maintenance chromosome flexible hinge domain containing 1 (SMCHD1) [17] or the DNA methyltrasferase 3B (DNMT3B) genes [18]. Recently, mutations in ligand-dependent nuclear receptor-interacting factor 1 (LRIF1), which may facilitate the interaction of SMCHD1 with chromatin, have also been associated with FSHD [19]. Interestingly, some FSHD2 individuals carry a shorter but normal D4Z4 repeat array than the average size in the control population [20], often associated with an earlier disease onset and more rapid progression. 

Today, there is no cure available for FSHD patients. The majority of current treatments aim to either enhance patients’ quality of life or slow disease progression, including physical therapy, low impact physical exercise, reduction of pain and fatigue, or surgery such as the scapula fixation procedure. Various therapeutic approaches have been already evaluated in FSHD patients including albuterol, a beta-2 receptor agonist [21,22], creatine monohydrate supplementation to enhance muscle performance [23], ATYR1940 to influence T-cell activation at the tissue level to promote healthier muscle (clinical trial number NCT02603562), and supplementation with antioxidant vitamins and minerals [24] but the results have been disappointing. More recently, Acceleron announced the discontinuation of its Phase 2 trial of ACE-083, a myostatin inhibitor, because it did not achieve statistically significant improvements in functional endpoints relative to placebo (clinical trial number NCT02927080). One Phase 2 trial, using Losmapimod (a selective p38α/β mitogen activated protein kinase (MAPK) inhibitor) is ongoing (NCT04003974). 

In this review, we will discuss the different DUX4-specific therapeutic strategies that have been considered in FSHD. The identification of *DUX4* has also led to the development of various animal and cell models, ranging from immortalized FSHD muscle cells to xenograft models, each having advantages and disadvantages over the others. 

## 2. Therapeutic Strategies in FSHD Targeting DUX4

**Figure 1:**
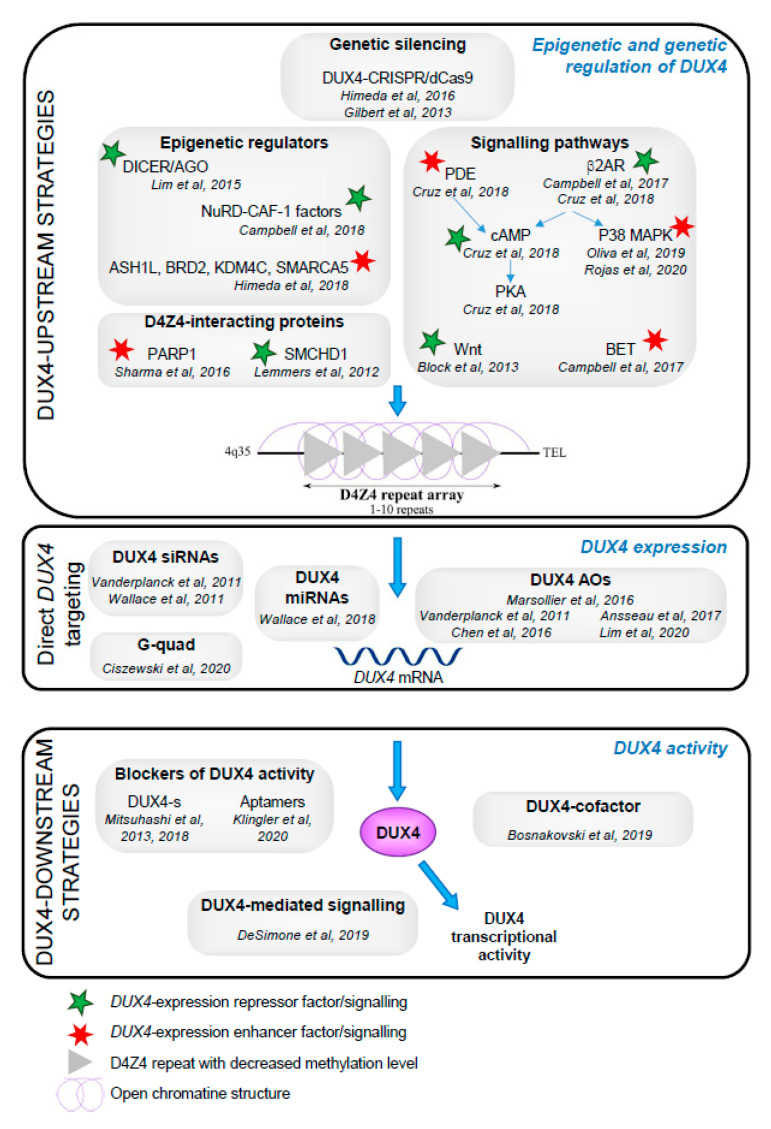
Graphical summary of therapeutic strategies targeting DUX4 in facioscapulohumeral muscular dystrophy (FSHD). Diagram summarizing the different therapeutic strategies discussed in this review. Targeting DUX4-mediated toxicity in skeletal muscle was achieved by (1) genetically or epigenetically regulating *DUX4* expression, (2) directly targeting *DUX4* transcript by RNAi or AOs-based approaches, and (3) inhibiting DUX4 activity. Numerous studies have tried to identify proteins and signaling pathways involved in regulating *DUX4* expression (top section of the diagram). These factors had either positive or negative effects on gene expression. The green star represents DUX4-repressors while the red star corresponds to enhancers of *DUX4* expression. The characteristic chromatin relaxation is illustrated in light purple and the D4Z4 hypomethylation status is depicted by the light grey triangles.

Developing a pharmacological treatment for FSHD patients has proven difficult, but since the identification of DUX4-mediated toxicity in muscle from patients, researchers are now focusing on developing therapeutic strategies targeting DUX4. Several approaches have now been considered and include (1) regulating *DUX4* expression to return to an asymptomatic state by correcting the epigenetic signature of the D4Z4 region or targeting the transcription of *DUX4*, (2) inactivating or destroying *DUX4* mRNA, and (3) blocking DUX4 protein activity to reduce toxicity associated with DUX4 target genes and downstream effects. This section aims to introduce and summarize the different therapeutic approaches targeting *DUX4* expression that have been considered for further treatment trials. A summary of the different therapeutic strategies targeting *DUX4* is presented in the Figure 1.

### 2.1. Upstream Approach: Targeting DUX4 Transcription 

As DUX4-toxicity is associated with its full length isoform expression in skeletal muscle [25], directly targeting *DUX4* expression to achieve an asymptomatic state in FSHD patients has been carried out by restoring the epigenetic repression of the D4Z4 region or targeting *DUX4* expression at its transcriptional level [26,27,28,29]. 

• Restoring the repressed epigenetic state of D4Z4

Both FSHD1 and FSHD2 patients are characterized by pathological epigenetic changes in the D4Z4 region, a critical dysregulation causing abnormal *DUX4* and *DUX4*-target gene expression in muscle fibers (for review see [1]). Correcting the epigenetic profile to regulate DUX4-mediated toxicity is an appealing strategy for therapeutic purposes and several studies have demonstrated that the epigenetic repression state of *DUX4* is indeed a reversible mechanism. In order to restore the epigenetic silencing of *DUX4,* epigenetic factors known to be associated with the D4Z4 locus have been specifically targeted [30]. In this study, several epigenetic regulation factors including ASH1L, BRD2, KDM4C, and SMARC5 have been silenced in primary FSHD muscle cells using either a shRNA-mediated RNA interference technique or a modified dCas9-KRAB silencing approach. The knockdown of these epigenetic regulators increased chromatin repression at the D4Z4 array and the expression of *DUX4* and *DUX4*-target genes *TRIM43*, *ZSCAN4*, and *MBD3L2* were efficiently reduced, although the epigenetic factors expression levels were only slightly diminished. 

Another approach is to boost *DUX4* silencers that bind the D4Z4 array. Recently, two D4Z4-binding factors NuRD (nucleosome remodeling deacetylase) and CAF-1 (chromatin assembly factor 1), were identified as mediators of *DUX4* chromatin repression [31]. As both complexes have been shown to silence the *DUX4* gene via their binding to the D4Z4 repeat, enhancing their repressing activity represent a potential therapeutic strategy. 

Altogether, these study suggest that rectifying the chromatin relaxation and restoring the epigenetic state at the D4Z4 locus could contribute to a considerable *DUX4* expression silencing.

• Reducing *DUX4* transcription levels

In addition to chromatin regulators, various signaling pathways regulate *DUX4* expression and constitute key therapeutic targets to reduce DUX4-mediated pathogenicity. The screening of chemical libraries identified bromodomain and extra-terminal (BET) bromodomain inhibitors and agonists of the beta-2 adrenergic receptor [26], as well as phosphodiesterase (PDE) inhibitors [32] as repressors of *DUX4* expression. Β2AR agonists and PDE inhibitors that induce increased cellular cAMP levels and PKA-dependent or -independent mechanisms were proposed [26,32]. The likely mechanisms to repress *DUX4* are mediated by lysine deacetylation (but not DNA methylation) for the BET inhibitors and through the beta-2 adrenergic receptor for the beta-adrenergic agonist compounds [26]. The screening of molecules that could interfere with the β2 adrenergic signaling allowed the identification of p38α and p38β MAPKs as suppressors of *DUX4* expression in FSHD myotubes and myoblasts and animal models by a mechanism that still needs to be elucidated [33]. These data support the repurposing of existing clinical p38 inhibitors as potential therapeutics for FSHD and interestingly, the treatment of xenografted mice [34] with Losmapimod, a well-known p38 inhibitor, resulted in dose-dependent decreases in mRNA levels for *DUX4* and *DUX4* target genes [33]. Losmapimod, initially developed by GlaxoSmithKline for the treatment of cardiovascular diseases, but now exclusively in-licensed from GSK by Fulcrum Therapeutics, is currently under evaluation in a Phase 2 clinical trial in FSHD patients (NCT04003974). 

The importance of Wnt signaling in *DUX4* repression has also been established in FSHD muscle cells [27]. Activating Wnt signaling pathway reduces *DUX4* expression whereas silencing Wnt pathway signaling mediators activates *DUX4* expression in primary human muscle cells [27].

*DUX4* promoter binding proteins such as PARP1 have also been identified and studied for their role in *DUX4* transcription. Inhibiting PARP1 in FSHD myoblasts suppresses *DUX4* and DUX4 target gene expression [28]. Another approach is the development of a CRISPR/dCas9-mediated transcriptional inhibition that targets the D4Z4 array and alters the chromatin to repress expression of *DUX4* [29]. Using a single sgRNA targeting the dCas9-KRAB [35] to the *DUX4* promoter or exon 1, a 2-fold reduction of endogenous *DUX4-fl* level was achieved in FSHD myocytes. Finally, siRNA targeting the promoter region induced a DICER/AGO-dependent epigenetic transcriptional silencing [36].

Recently, G-quadruplex (GQ) structures, which are formed in DNA or RNA that are rich in guanine through the formation of Hoogsten hydrogen bonds between bases (for review see [37]), were identified within the enhancer, promoter, and *DUX4* transcripts [38]. Berberine, an alkaloid that can be extracted from a variety of plants and previously shown to bind G-quadruplex DNA and RNA structures [39,40], was used to inhibit DUX4 expression. A reduction of *DUX4* mRNA was observed in FSHD cells but not *in vivo* after a local injection of an adeno associated virus-DUX4 (AAV-DUX4) followed by repeated intraperitoneal injections of Berberine [38]. However, *in vivo*, a reduction of the DUX4 protein was observed. The muscle specific force was increased in the presence of the berberine but surprisingly, was also associated with a reduction in the mass of the treated muscle. The exact mechanism of action is unknown and the Berberine could interfere with mRNA transcription or translation of *DUX4*. One major limitation of this approach is the impossibility to target specific GQs within the genome. 

Altogether, these studies suggest that upstream of *DUX4* transcription, specific factors from multiple signaling pathways regulate *DUX4* expression either via direct binding to a *DUX4* promoter, or activation of a transcription factor negatively regulating *DUX4* expression. 

### 2.2. Targeting DUX4 mRNA

There are two distinct types of therapies that target mRNA: Double strand molecules that are dependent on the endogenous microRNA pathway and single-stranded antisense oligonucleotides (ASOs), which trigger different outcomes altering mRNA processing and stability. Both eventually lead to mRNA silencing, and both approaches have been envisaged to decrease *DUX4* expression. 

• RNAi-based methods

siRNAs and microRNAs are the most popular small RNAs used to silence gene expression. siRNA mediates gene silencing through sequence-specific cleavage of 100% complementary mRNA, whereas miRNA induces mRNA degradation and inhibits the translation of imperfectly complementary targets. In FSHD, both siRNA and miRNA silencing techniques have been developed to prevent the translation of *DUX4* mRNA into DUX4 protein and inhibit its toxic downstream effects. A significant reduction of *DUX4* at both mRNA and protein levels was achieved after the transfection of siRNA against *DUX4* in FSHD primary myotubes [41]. A decreased expression of the *DUX4*-network genes was also observed. Another group designed several miRNAs to specifically target *DUX4* (miDUX4) and their efficacies were assessed in an *in vivo* mouse model expressing DUX4 [42,43]. Following miDUX4 injection, DUX4-expressing muscles were protected from histopathological damage, including muscle degeneration and the amounts of DUX4 protein and *DUX4* mRNA were significantly decreased. Together, these studies suggest that small RNAs such as siRNA and miRNA targeting *DUX4* mRNA have a significant impact on DUX4 protein translation that could provide interesting therapeutic advantages. 

• Antisense oligonucleotide-based methods

Antisense oligonucleotides are short sequences of about 13–25 nt single strand DNA or RNA sequences designed to alter the original function of the target mRNA through an array of different mechanisms including 5′ cap formation inhibition, translation prevention through steric blockade, splicing alteration, and more commonly RNaseH-mediated RNA degradation via complementary base-pairing to the target mRNA (for review see [44]). The use of AOs in FSHD pathology was proposed almost 10 years ago by the Belayew laboratory [41]. Indeed, the treatment of FSHD myotubes with 2′-O-methyl bases on a phosphorothioate backbone, targeting splice sites of exons 2 and 3, reduced endogenous *DUX4* by up to 50% and reduced FSHD marker expression [41,45]. Later, these AOs were evaluated in wild type mice co-injected with an AAV-mediated DUX4 expression and vivo-PMO (phosphorodiamidate morpholino oligomer) targeting exon 3 [45]. A 30 fold reduction in *DUX4* mRNA was observed in the presence of the vivo-PMO. 

Since the development of third generation AOs (phosphorodiamidate morpholino oligomer (PMO)), 2 independent teams have developed PMOs targeting the polyadenylation signal site in the DUX4 transcript [46,47]. Both studies demonstrated the efficacy of morpholino-mediated *DUX4* transcription inhibition in a dose-dependent manner in primary FSHD myoblasts [47], immortalized FSHD clones [46,48], and in a xenograft mouse model [47,49]. All *in vitro* and *in vivo* FSHD models confirmed a significant decrease of *DUX4-fl* transcript expression levels associated with an inhibition of DUX4-fl activity, as multiple target genes (*TRIM43*, *MBD3L2*, and *ZSCAN4*) were also efficiently down regulated [46], thereby confirming the therapeutic properties of AOs and morpholinos in correcting DUX4-mediated toxicity in skeletal muscle. More recently, a locked nucleic acid (LNA) gapmer antisense oligonucleotide was developed to silence *DUX4* [50]. LNA gapmers present a central DNA gap flanked by LNA stretches to increase the binding affinity to the target RNA. Unlike PMOs and 2′-O-methyl, when the gapmer is hybridized to its target RNA, the RNase H protein is recruited to cleave it, leading to gene silencing. *In vitro*, a ~100% knockdown of *DUX4* was achieved at 100 nM and RNA sequencing showed a decreased expression of FSHD-associated genes. The efficacy of the gapmer LNAs were evaluated in the uninduced FLExDUX4 mouse that expresses *DUX4* at very low levels, so low that the DUX4 network genes are not induced (see below for the mouse characteristics). The authors reported a 70% knockdown of *DUX4* mRNA. 

### 2.3. Downstream Approach: Targeting DUX4-fl Protein Toxicity 

Targeting DUX4 post-translationally is also a strategy that has recently been developed to either block DUX4 protein activity or repress its downstream toxic signaling pathways.

• Blocking the activity of the DUX4 protein

*DUX4* is a double homeobox retrogene and 3 main *DUX4* isoforms have been characterized (for review see [13]). Two lead to the full length protein (DUX4-fl) that is a toxic transcription factor, whereas the third one leads to a non-pathogenic protein (DUX4-s). In the *DUX4-s* isoform, an alternative donor splice site located in first exon is used [12], leading to a truncated form of DUX4, lacking the transactivation domain that is located in the C-terminal part of the protein, but carrying the 2 DNA-binding domains [51]. Consequently, DUX4 and DUX4-s recognize and bind the same DNA binding motifs but DUX4-s is unable to act as a transcription factor and does not activate gene expression [52]. One group took advantage of this particularity to develop a dominant negative form of DUX4 based on DUX4-s over-expression. In a zebrafish model of FSHD, co-injection of both short non-toxic (DUX4-s) and full length (DUX4-fl) of DUX4 protein led to a decrease in the phenotypical abnormalities observed in animals singly injected with DUX4-fl [53]. DUX4-s acted as an agonist of DUX4-fl, preventing its binding to DNA and ultimately blocking its transcriptional activity [51]. These novel discoveries demonstrate that DUX4-s acts as an inhibitor that competes with DUX4-fl to reduce its toxicity. 

Molecules that binds and sequester DUX4 with high specificity also represent an attractive strategy to block DUX4 cytotoxicity in skeletal muscle. Aptamers are DNA- or RNA-based molecules with the ability to bind highly specific targets including proteins, peptides small molecules, and even whole cells. Using aptamers to specifically target a transcription factor, such as DUX4, represents an interesting technique to prevent transcription factor-DNA binding and regulate DUX4 activity. In a recent paper [54], DUX4-specific aptamers were designed to bind DUX4 protein with high affinity. The aptamer structure had important specific structural elements in addition to a DUX4 binding site that increased its binding activity to DUX4. These structures include an optimized size length and sequence bulge loop and hairpin-like domains. 

Targeting DUX4 cofactors or downstream consequences may also be a therapeutic approach for FSHD. Two proteins with histone acetyltransferase (HAT) activity—p300 and cAMP response element-Binding Protein (CREB) binding protein (CBP)—are factors interacting with the C-terminal domain of DUX4 and more importantly are recruited to DUX4-target protein binding sites [55]. Recruitment of HATs to DUX4-target genes results in further acetylation and expression of target genes. Targeting the DUX4/p300 and/or DUX4/CBP interaction or the HAT activity of these DUX4-binding factors constitutes a potential check point to control DUX4 activity. iP300w is a compound targeting HAT activity and in a recent study, iP300w was used to regulate DUX4 cytotoxity in skeletal muscle [56]. Muscle cells expressing DUX4 treated with the iP300w molecule displayed a decrease in DUX4-mediated toxicity and *in vivo*, the treatment of a DUX4-expressing mouse induced a decreased expression of DUX4 target genes. However, a few DUX4 targets, such as ZSCAN4, are apparently insensitive to iP300w, indicating that an alternative regulation may occur. This is important because the DUX4 targets that are directly implicated in FSHD pathogenesis are not well-characterized and *DUX4*-network genes are often used as a biomarker of DUX4 activity, regardless of their physiological role and impact. This study also highlights that compounds disrupting the DUX4-EP300 or -CBP interaction would have potential therapeutic benefits. 

• Targeting DUX4-mediated signaling pathways

Lastly, DUX4 pathogenic activity in skeletal muscle can be reduced by targeting DUX4-mediated downstream signaling pathway toxicity. It was recently proposed that the hyaluronic acid (HA) pathways mediates DUX4 myogenic toxicity [57]. DUX4 expression in skeletal cells induced an abnormal accumulation of HA, which is associated with several abnormalities mediated by DUX4, such as the induction of dsRNA foci or FUS aggregation. Treatment of the human myoblasts MB135-DUX4i with 4-methylumbelliferone (4MU), which is a competitive inhibitor of HA biosynthesis, prevents these DUX4-mediated nuclear pathologies and reduces DUX4 cytotoxicity. Although 4MU had very low impact on *DUX4* expression, *DUX4* target candidates such as *LEUTX* and *MBD3L2* were strongly down regulated. 

## 3. Cell Lines and DUX4 Animal Models

Since the discovery of the role of *DUX4* in FSHD pathology, the development of cell and animal models have been the center of attention to study the DUX4-mediated toxicity in FSHD muscles and more importantly to evaluate therapeutic and pharmaceutical strategies targeting DUX4. The first obstacle was that *DUX4* encoded by the D4Z4 repeat is a primate-specific macrosatellite and needs to be exogenously provided to produce an animal model [58]. But the main hurdle in developing a DUX4-expressing animal model was that the full length isoform of *DUX4*, which is responsible for muscle pathology in FSHD patients [25,59], is so cytotoxic when expressed in somatic tissues that animals die during development. Leaky expression of *DUX4* in animal models was the major problem to overcome and a stringent regulation of DUX4 expression level was thus required. 

Another important point in the creation of an animal model was to recapitulate DUX4 and FSHD biology. Indeed, FSHD patients are characterized by a stable *DUX4-fl* mRNA expression in a low proportion of muscle cells driving a cascade of toxic signaling pathways resulting in muscle disease [41,60,61,62]. Patients also present an atrophy of specific groups of muscles, non-muscular symptoms, and a left-right asymmetry of muscle symptoms. Altogether, these characteristics should be taken into consideration in the DUX4-based FSHD animal models, but none of the models fully encompasses them. All present advantages and disadvantages, depending on the scientific questions that are asked. Table 1 provides a summary of the models that can be used, depending on the therapeutic strategy used to target DUX4. 

In this section, we will discuss cell and animal models including mice, zebrafish, and drosophila DUX4 models that have been developed over the years. 

### 3.1. Cell Lines 

The first DUX4-expressing model was created by Bosnakovki et al. [63]. The authors generated an inducible cassette exchange in the mouse myoblast C2C12 cell line, which is regulated by the addition of doxycycline. DUX4 is expressed in a doxycycline-dependent manner associated with an enhanced sensitivity to stress-inducing compounds, impaired differentiation, and cell death. The principal limitation of this model is that DUX4 is expressed in a murine cell line. 

A few human cell lines were also developed. Adult primary myogenic cells can be directly isolated from muscle biopsies, but have a limited lifespan and senescence after approximately 25 rounds of division in tissue culture. They are also often heterogeneous in terms of cell type. To overcome these limitations, immortalized cells were generated after transduction by two retroviruses carrying *hTERT* and *Cdk4* [48]. Clones isolated from the transduced population express DUX4 and *DUX4* downstream genes after differentiation and do not present any dysregulation of normal myogenic processes [48,75]. These vectors were used to immortalized cells from mosaic patients, thus generating isogenic D4Z4 contracted and non-contracted clones [48]. 

More recently, a doxycycline-inducible *DUX4* in immortalized myoblasts (iMB135) was generated using an optimized *DUX4* cDNA with a reduced number of CpG sites but preserved protein sequence [59]. Indeed, the 130 CpG sites present in the DUX4 open reading frame (ORF) may be subjected to DNA methylation, leading to low *DUX4* expression. The authors showed that clones carrying the *DUX4* optimized sequence show high transgene expression whereas surprisingly, none of the five wild-type *DUX4* clones showed induction of *DUX4* mRNA. The optimization of the sequence presents an advantage in terms of DUX4 expression but complicates the design of AOs since the sequence is not conserved. 

### 3.2. Mouse Models 

• AAV-DUX4 (2011–)

One of the earliest FSHD mice models produced goes back to the AAV6-DUX4 overexpressing mice generated by Wallace et al. [64]. *DUX4* was cloned under the control of the CMV promoter and 3 × 10^10^ DRP AAV6-DUX4 were intramuscularly injected in 6- to 8-week-old mice. Much muscle damage including cell infiltration, fibre degeneration, and TUNEL-positive nuclei was visible, demonstrating the toxicity of *DUX4* expression in muscle cells. *DUX4* mRNA expression in skeletal muscle was validated by *DUX4* quantification by RT-PCR and immunolabelling analysis. Since then, several studies aiming at reducing the DUX4-mediated toxicity have been tested following the design of this mouse model [38,42,43]. 

• D4Z4-2.5 (2013)

Both D4Z4-2.5 and −12.5 mouse models carry D4Z4 units at mouse chromosome 17 but differ in the number of copies (2.5 and 12.5 respectively). These units were derived from a pathogenic permissive contracted allele from a FSHD1 patient [65]. Both mice expressed *DUX4* in their germline. Interestingly the D4Z4-12.5 show that both proximal and distal D4Z4 units were highly methylated (60–90%) whereas the D4Z4-2.5 was relatively hypomethylated (10–20%). The D4Z4-2.5 mouse expressed *DUX4* mRNA in skeletal muscles, but these levels were low and varied considerably between gender-matched littermates. Expression of *DUX4* in non-muscle tissue was also reported, including neurological tissues and eyes. *DUX4* expression was also reported in myogenin-negative cells, illustrating the inefficient repression of DUX4 in somatic cells. Overall the D4Z4-2.5 mice model failed to reproduce a FSHD phenotype with no apparent muscle damage except for the eye phenotype and incorrect *DUX4* tissue expression. The authors also stated that more than 450 injections were required before producing a functional line, making the D4Z4-2.5 a difficult model to design and inadequate for scalable therapeutic trials. 

• iDUX4(2.7) (2014–2016)

To be able to study DUX4 expression *in vivo*, the mouse model iDUX4(2.7) was designed based on a doxycycline-inducible DUX4 expression [66,67]. A 2.7kb *DUX4* gene fragment, containing *DUX4* ORF and the 3’UTR sequence with an additional SV40 poly(A) signal present in the vector, was integrated into the X-chromosome of animals. The amount of *DUX4* mRNA was overall barely detectable in individuals and DUX4 protein expression could not be quantified or distinguished from background, and a leaking baseline DUX4 expression was observed without doxycycline administration [66]. Slight muscle damage was reported by the authors, as iDUX4(2.7) mice muscles were smaller. They recorded a decrease in animals’ strength but this was correlated with mouse body size. Contrary to the D4Z4-2.5 model, the iDUX4(2.7) mice showed greater muscle damage, but still displayed a faint muscle dystrophy phenotype. iDUX4(2.7) animals were characterized by complete demethylation profiles. Non-muscle features included loss of high-frequency hearing and a similar eye phenotype to the D4Z4-2.5 mice was observed [67]. Critically, major issues were raised with the iDUX4(2.7) model regarding male-specific short life-span or early lethality. Subsequently, the authors discovered significant read through of the endogenous *DUX4* poly(A) and hypothesized that the use of the downstream SV40 p(A) may make the transgene highly toxic [69]. Female animals were also unsuitable for analysis due to the female-specific biased X-inactivation and only required for breeding. On a more positive note, even if very low *DUX4* transcripts were detectable, the iDUX4(2.7) model showed that barely measurable amounts of *DUX4* were sufficient to induce toxicity and high level of lethality in mice, suggesting that tight DUX4 expression regulation had to be taken into careful consideration to design an acceptable animal model. 

• D4Z4-2.5/Smchd1MommeD1 mice (2018)

Following issues encountered with their D4Z4-2.5 mice model which 1) expressed low levels of *DUX4* transcripts and protein in skeletal muscle and 2) did not exhibit a muscle phenotype [65], the authors decided to use this model to consider the role of Smchd1 in D4Z4 chromatin structure, DUX4 expression, and disease severity [68]. A bi-transgenic mouse model was designed by crossbreeding their previous D4Z4-2.5 mice with a Smchd1 haploinsufficient strain (Smch1MommeD1 mice). Similarly, very low levels of *DUX4* mRNA expression were measured in the D4Z4-2.5/Smchd1MommeD1 mouse model and a high variability was detected among different individuals. Moreover, animals suffered from higher levels of *DUX4* expression in non-muscle tissues such as in skin tissue and thymus. The newly designed D4Z4-2.5/Smchd1MommeD1 mice still lacked a FSHD phenotype with no muscle damage and *DUX4* misexpression in non-muscle tissues. Altogether, these studies demonstrated that the D4Z4-2.5 and the bi-transgenic mouse models were not suitable for modeling DUX4-mediated toxicity in skeletal muscle, nor to test pharmaceutical molecules or other therapeutic strategies. However, these animals still remain interesting models to study the epigenetic signature of the D4Z4 repeat and its defective repression in FSHD. 

• iDUX4pA-HSA (2017–2020)

The major problem that needed to be overcome in designing an appropriate FSHD mouse model was the DUX4-induced toxicity and lethality observed in animals. Controlling *DUX4* expression levels was therefore a critical feature in developing an animal model. By designing a conditional and titratable model using the endogenous and low efficient *DUX4* polyadenylation signal, Bosnakovski et al. aimed at reducing the leaky DUX4 expression-mediated toxicity. An inducible mouse model was produced by knocking in a genomic fragment from the D4Z4 repeat belonging to the 4qA allele of a FSHD patient (including the *DUX4* ORF, 3′UTR including the *DUX4* p(A)) that was placed under the control of a doxycycline-inducible promoter called iDUX4pA [69,70]. The HSA-rTA driver allowed for a muscle-specific *DUX4* expression-induction in animals. Uninduced animals of both sex had a mild phenotype, ranging from body/muscle weight loss to extremely low *DUX4* mRNA and target expression levels. Females developed milder symptoms than males. Doxycyline treatment was responsible for progressive dystrophic changes including heterogeneous myofibre size, increased cell death, and cell infiltration. DUX4 expression in muscle nuclei was restricted to a small fraction with a sporadic expression pattern and several DUX4 candidate genes were also upregulated. Altogether, the authors designed a viable mouse model characterized by a muscle-specific and inducible DUX4 expression. They recently used this same animal model to create a chronic long-term muscle disease model [70]. Rather than daily injection, doxycycline was chow-administered for up to six months. Similarly, animals developed a slow progressive muscle pathology with muscle atrophy and a significant decrease in force-generation capacity was observed. Signs of muscular dystrophy also progressed during doxycycline administration, with increased inflammatory and fat infiltration. The DUX4 expression pattern remained sporadic and few myofibres tested positive for DUX4. A transcriptomic analysis on whole muscle revealed that muscles from the iUDX4pA-HSA mouse model recapitulate the gene expression signature found in MRI-guided human FSHD muscle biopsies, including genes associated with cell-death signaling pathways as well as *DUX4* target genes The iDUX4pA mouse model has been used to develop a therapeutic strategy by the team, showing that the p300-inhbiting molecule ip300w efficiently decreased DUX4-mediated toxicity in skeletal muscle [56].

• ACTA1-MCM-FLExDUX4 (2018–2020)

The Jones laboratory generated a double transgenic mouse line called ACTA1-MCM-FLExDUX4 that conditionally express DUX4 in muscles after tamoxifen injection [71]. This line was generated after crossing the FLExDUX4 with the ACTA1-MerCreMer mouse. The FLExDUX4 mouse carries locked *DUX4* exons 1 to 3, including the *DUX4* poly(A) signal that was inserted in the ROSA26 locus in the antisense orientation to the Rosa26 promoter, thus preventing its correct transcription. However, the authors noted that a sense polyadenylated *DUX4-fl* is produced in skeletal muscle from the inverted transgene, whereas both liver and skin expressed shorter forms of *DUX4*, distinct from *DUX4-s*. But no muscle phenotype was observed, no DUX4 protein was revealed after immunostaining, and there was no activation of the murine *DUX4* target gene *Wfdc3*. Despite the very low level of *DUX4* expression and the absence of downstream effects, this mouse model was recently used to assess antisense LNA gapmer efficacy [50]. 

An expression of the DUX4 target genes can be achieved when the expression of the transgene is under the control of the Rosa26 promoter. Indeed, the transgene is flanked by heterologous loxP sites that recombine in the presence of CRE recombinase. The FLExDUX4 model was crossbred with a muscle specific ACTA1-Mer-cre-Mer mouse, resulting in the bi-transgenic ACTA1-MCM; FLExDUX4 model. Tamoxifen allowed the translocation of the CRE protein from the cytoplasm to the nucleus, allowing the inversion of the transgene to the sense orientation. The authors observed a tamoxifen dose-dependent recombination rate, leading to different expression of several *DUX4* downstream genes associated with many abnormal physiological signs including cell death, immune response, increased centralized nuclei, and lower specific force [76]. Skeletal muscles underwent higher levels of myofiber regeneration as well as fibrosis. The most severely affected mice rapidly lost their motility. This bi-transgenic mouse thus constitutes an interesting animal model to study the mosaic expression pattern of DUX4-fl in skeletal muscle. The FLExDUX4 mice is particularly appealing as numerous studies can be performed from short- to long-term (several months) analyses, as different levels of phenotype severity can be achieved.

• TIC-DUX4 (2018)

Giesige et al., generated a tamoxifen-inducible Cre-DUX4 double transgenic mouse line called TIC-DUX4 that conditionally expresses *DUX4* in muscles after tamoxifen injection [43]. Similar to the FLExDUX4 mouse, the TIC mouse carries the *DUX4* gene and pLAM sequence, but has an additional downstream poly(A) from the canonical bovine growth hormone (BGH). A PGK-neomycin-resistance cassette flanked by LoxP sites was cloned between the ROSA26 promoter and the *DUX4* ORF, preventing *DUX4* transcription in the absence of CRE. The mouse also carries a HSA-mER-CRE-mER transgene, allowing the transcription of CRE only in the muscle. The authors observed a tamoxifen dose-dependent muscle damage at molecular, histological, and functional levels that was associated with *DUX4* expression. Interestingly, 1.5-year-old uninduced mice show some dystrophic featured, including muscle fibers with centrally located nuclei and sporadic loci of immune cell infiltration. There was no difference in body weight between old TIC and wildtype mice but TA muscles were ~30% smaller in the TIC animals. Altogether the TIC-DUX4 mice model, in association with an adequate tamoxifen dosing administration, permitted long-term *in vivo DUX4* expression and slow and progressive phenotype development. This mouse cannot be used to evaluate therapies targeting the poly(A) region due to the presence of the downstream BGH poly(A) signal. 

• Xenografts (2014–2019)

Skeletal muscle engraftment is a procedure regularly studied in disease-associated muscle damage such as muscle dystrophy-induced atrophy and wasting (reviewed in [34]). The term engraftment or “xenograft” refers to the transplantation of biological material (that could be specific tissue or cells in suspension), from a donor of one species into the recipient of another species. Xenograft models have been largely used to analyze therapeutic strategies to correct muscle diseases. For the purpose of this review, the term xenograft will exclusively refer to the engraftment of human muscle tissues or cells into a mouse host. Xenografts models have been produced in immunodeficient mice hosts that have sustained muscle injury to prepare the niche to seed the donor cells within the recipient muscle. The human muscle graft is then directly implanted following a surgical procedure, or muscle cells are injected intramuscularly. Depending on the study, engraftment can last up to several months until further analyses are performed. One limitation is the inability to assess whole muscle functions such as treadmill endurance. Also, because xenografts are made in immunodeficient mice, this mouse model cannot be used to study the effects of DUX4 on the immune system. 

The first xenograft model using FSHD-derived muscle tissues was designed by Zhang et al. [49]. Their mouse host was the female immunodeficient NOD-Rag mice and the tibialis anterior and extensor digitorum longus muscles were surgically removed and small segments of unaffected control or patient muscle biopsies were engrafted. Grafts become innervated, vascularized, and displayed functional contractility. Compared to healthy control-engrafted mice, FSHD muscle biopsy-engrafted hosts displayed *DUX4-fl* expression associated with an upregulation of DUX4-target genes such as *MBD3L5*, *TRIM43*, and *ZSCAN4*. Using affected muscle biopsies from FSHD patients grafted into hindlimbs of NOD-Rag immunodeficient mice, a morpholino designed to target the polyadenylation signal of *DUX4* mRNA efficiently reduced levels of the toxic DUX4-fl and its downstream genes *MBD3L5* and *ZSCAN4* [47]. However, this model retains some limitations, as only very small numbers of human muscle biopsies and small amounts of tissue are available, so only a limited number of mouse models can be generated, which makes this model unsuitable for large scale therapeutic treatments trials. 

To overcome this limitation, another xenograft model has been produced using immortalized cells [72,73]. The team used male immunodeficient NOD-Rag mice and applied a combination of X-ray irradiation and myotoxins to remove host muscle progenitors and promote the regeneration of muscle fibres of donor origin. Human muscle precursor cells were collected from a FSHD patient and injected in the mouse anterior tibial compartment. To enhance muscle regeneration, the team applied the intermittent neuromuscular electrical stimulation (iNMES), that improved the quality of the graft and the number of human skeletal fibres [72]. This model was characterized by increased *DUX4* and DUX4-target mRNA expression compared to control xenograft models [73]. DUX4 expression was restricted to limited myonuclei, as in the sporadic and transient DUX4 expression characteristically observed in FSHD patients [61]. Importantly, the FSHD pathological epigenetic signature is conserved in this xenograft model with similar hypomethylation profiles exhibited in patients. In the paper published by Oliva et al. [33], a clinical p38 inhibitor was successfully tested in a xenograft model of FSHD using FSHD-derived myoblasts. The authors demonstrated that inhibiting the β2-adrenergic-associated p38 kinase in the FSHD xenograft mice model decreased *DUX4*- and DUX4 target genes expression levels in a dose dependent manner [33]. 

### 3.3. Drosophila Model

To better understand the mechanisms underlying DUX4 toxicity in skeletal muscle, Jones et al. designed a *DUX4*-overexpressing transgenic drosophila [74]. Indeed, compared to vertebrate models, drosophila have a short life cycle of 8–14 days, produce a large number of embryos and can be easily genetically modified. Transgenic drosophila were successfully generated by placing *DUX4-fl* expression under the control of the GAL4-upstream activation sequence. However, developmental lethality was conserved in Drosophila, as ubiquitous DUX4 expression in adult tissues led to the generation of non-viable animals. Similarly, when *DUX4* expression was specifically targeted to skeletal muscles, the authors were unable to obtain viable flies. Once DUX4 expression was restricted to the eye during development, a few viable drosophila were produced, probably due to the low ectopic and toxic expression of DUX4-fl in the other tissues.

### 3.4. Zebrafish Models 

Zebrafish have been an appealing animal model in muscle diseases for multiple years (see review [77]). Because their embryos are transparent and muscle development is rapid, those doing developmental studies have been particularly interested in this model. More importantly, zebrafish body musculature is almost identical to human skeletal muscles and they share similar gene-associated muscle diseases. In the context of FSHD, several zebrafish models have been produced [53,64]. A DUX4-zebrafish animal model has been created using the Tol2 transposon technique and the muscle-specific MHCK7 promoter to drive *DUX4* expression [64]. These transgenic zebrafish over-expressing DUX4 developed whole body malformations and high levels of muscle histopathology. A second zebrafish model has since be developed by an independent team. Fertilized zebrafish eggs were injected with copies of the human *DUX4* mRNA [53]. Similarly, DUX4-expressing zebrafish developed muscle development dysregulation, with asymmetrical phenotypes affecting face muscles and fins. Together, these models illustrate that the misexpression of human DUX4 in zebrafish mimic some clinical features of FSHD patients, however animals suffered extended muscle pathology, which is not characteristic of specific muscle weakness observed in FSHD patients. 

## 4. Limitations and Hurdles

Several hurdles still need to be overcome for the development of an efficient therapy that targets DUX4. Skeletal muscle is the largest organ in the body and a systemic delivery approach may be required to treat the disease. Most of the limitations are not FSHD/DUX4 specific. For example, a strategy targeting *DUX4* mRNA using an antisense oligonucleotide will suffer of the same limitations as in other neuromuscular diseases, such as the low intracellular delivery, distribution, long term toxicity, or potential immunogenicity that may vary according to the type of chemical modifications introduced on the nucleic acids (for review see [78]). Similarly, off-target effects should be investigated when molecules targeting broad cellular mechanisms such as signaling pathways, epigenetic regulators, or DUX4 co-factors. The use of AAV-based therapies may have shown some limitations. For example, pre-existing immunities to AAV are often found in humans, excluding a large proportion of patients from enrolment [79] and AAV gene transfer triggers an immune response to the AAV capsid, thus preventing the re-administration of the vector. Many laboratories are working on these limitations and proposed solutions include the AAV-specific depletion of neutralizing antibodies using a plasmapheresis column [80], the administration of IgG-cleaving endopeptidases to decrease anti-AAV antibody titers [81], the chemical modification of the AO to improve its pharmacokinetic and pharmacodynamic properties, and the conjugation to antibody or peptide to ameliorate muscle uptake. 

Some therapeutic hurdles are specific to FSHD/DUX4. As *DUX4* is expressed at very low levels, in bursts and in a very limited number of nuclei [82], one important question is: How much DUX4 diminution is required for clinical efficacy? Moreover, DUX4 spreads along the muscle fibers [60] raised the question of how many nuclei have to be targeted to counteract pathological spreading. Strategies targeting the DUX4 protein may have an advantage over those targeting *DUX4* mRNA because they may be able to trap the protein during its cytoplasmic journey, independently of the nuclei transcribing *DUX4*, whereas antisense approaches needs to target most, if not all, the nuclei in the muscle fiber to suppress *DUX4* expression. Finally, since *DUX4* is already expressed during fetal muscle development [83], a crucial question concerns the therapeutic window for treatment. 

## 5. Conclusions 

The route to find a treatment for FSHD is still long and many efforts are being made to provide patients with a cure. Unfortunately, DUX4 is specific to humans and the different models created so far illustrate the difficulty encountered in developing a DUX4-expressing animal. Preclinical tests still remain a major condition to proceed with clinical trials and the design of a model that would recapitulate FSHD clinical signs is a major goal. Most laboratories focus on DUX4, as *DUX4* is currently the consensus disease gene, but other factors may participate in FSHD onset or progression such as *FAT1* [84,85] or DUX4c [86]. 

So far, several clinical trials have been designed for FSHD patients but most of them gave disappointing results. For example, as *DUX4* expression leads to increased susceptibility to oxidative stress, a cocktail of antioxidants were administrated in a double-blind trial to FSHD patients, but only a slight increase of the maximum voluntary contraction and endurance of the quadriceps were observed [24]. Another potential therapy was to inhibit a natural muscle growth inhibitor called myostatin. Two trials using two different myostatin inhibitors were conducted, but both were discontinued due to no significant improvements in functional endpoints relative to placebo [87,88]. Downregulation of the myostatin pathway in neuromuscular diseases may be part of the explanation for this low clinical efficacy [89]. In another trial, Albuterol, a β2-adrenergic receptor agonist, was used to increase muscle mass and strength [21,90] and the random double-blind, placebo-controlled trials did not show any muscle strength and function improvement despite its positive effect on muscle mass. Among the kinases activated by the β2-adrenergic-medated signaling pathway is p38 MAPK. Three studies highlighted the potential benefit of targeting p38 to decrease DUX4 expression [33,73,91] and Losmapimod, a selective p38α/β inhibitor, is now being tested in ongoing clinical trials. A randomized, double-blind placebo-controlled study is currently in phase 2 (NCT04264442) for FSHD1 patients. 

Other therapies targeting DUX4 are also in development/pre-clinical stages. These therapies are based on the use of antisense oligonucleotides, AAV-based approach, gene editing, or small molecules. One of the most critical points will be the identification of outcome measures of clinical efficacy. Several have been proposed such as clinical severity score (CSS) [92], the reachable work space (RWS) [93], and muscle MRI [94,95], or are under development such as the FSHD-COM [96]. While these techniques are informative, they suffer from several limitations as they are often costly, laborious, low through-put, or allow the assessment of a limited number of muscles. Tissue biomarkers could also be used to measure changes in *DUX4* expression or in the DUX4 network genes, but they are invasive (a muscle biopsy is necessary) and reflect the state of the muscle only at the time of sampling on a very limited portion of muscle. Importantly, there is no consensus either on which genes downstream of DUX4 reflect muscle heath, or if they can be used to monitor treatment efficacy. The development of blood circulating biomarkers are very important since they are not costly, are low-invasive, and can monitor drug response over time. Two studies described potential serum biomarkers by comparing FSHD and control individuals [97,98]. More recently, two independent groups reported that myostatin could be a quantifiable biomarker to monitor drug response in several neuromuscular diseases including Duchenne muscular dystrophy [99] and myotubular myopathy [100]. It was proposed that myostatin levels could be a clinically meaningful, reflecting the global muscle health biomarker in other neuromuscular diseases. Defining robust and reliable blood based biomarkers to monitor treatment over time in FSHD patients will be one of the most important limitations to overcome. 

## Figures and Tables

**Table 1:** Models that can be used depending on their therapeutic therapy.

Name of the Model		Therapeutic Strategies Targeting			Functional Read Out
		*DUX4* Promoter	*DUX4* mRNA	DUX4 Protein	
Cell lines	FSHD cells [48]	Yes	Yes	Yes	No
	DUX4 overexpression [59,63]	No	Yes	Yes	No
AAV-DUX4 [38,42,43,64]		No	Yes	Yes	ND
D4Z4-2.5 [65]		Yes	No	No	No
iDUX4(2.7) [66,67]		No	Yes *	Yes	Yes
D4Z4-2.5/Smchd1MommeD1 [68]		Yes	No	No	No
iDUX4pA [69,70]		No	Yes	Yes	Yes
ACTA1-MCM-FLExDUX4 [71]		No	Yes	Yes	Yes
TIC-DUX4 [43]		No	Yes *	Yes	Yes
Xenograft [49,72,73]		Yes	Yes	Yes	No
Drosophila [74]		No	No	No	No
Zebrafish [53,64]		No	Yes	Yes	Yes

*: presence of an additional downstream signal.
